# Correlated Inter-Domain Motions in Adenylate Kinase

**DOI:** 10.1371/journal.pcbi.1003721

**Published:** 2014-07-31

**Authors:** Santiago Esteban-Martín, Robert Bryn Fenwick, Jörgen Ådén, Benjamin Cossins, Carlos W. Bertoncini, Victor Guallar, Magnus Wolf-Watz, Xavier Salvatella

**Affiliations:** 1 Joint BSC-CRG-IRB Research Programme in Computational Biology, Barcelona Supercomputing Center - BSC, Barcelona, Spain; 2 Joint BSC-CRG-IRB Research Programme in Computational Biology, Institute for Research in Biomedicine – IRB Barcelona, Barcelona, Spain; 3 Department of Chemistry, Chemical Biological Centre, Umeå University, Umeå, Sweden; 4 Institució Catalana de Recerca i Estudis Avançats - ICREA, Barcelona, Spain; Max Planck Institute for Biophysical Chemistry, Germany

## Abstract

Correlated inter-domain motions in proteins can mediate fundamental biochemical processes such as signal transduction and allostery. Here we characterize at structural level the inter-domain coupling in a multidomain enzyme, Adenylate Kinase (AK), using computational methods that exploit the shape information encoded in residual dipolar couplings (RDCs) measured under steric alignment by nuclear magnetic resonance (NMR). We find experimental evidence for a multi-state equilibrium distribution along the opening/closing pathway of Adenylate Kinase, previously proposed from computational work, in which inter-domain interactions disfavour states where only the AMP binding domain is closed. In summary, we provide a robust experimental technique for study of allosteric regulation in AK and other enzymes.

## Author Summary

Most proteins contain several domains, and inter-domain motions play important roles in their biological functions. Describing the various inter-domain orientations that multi-domain proteins adopt at equilibrium is challenging, but key for understanding the relationship between protein structure and function. When more than two domains are present in a protein, correlated domain motions can be of fundamental importance for biological function. This type of behaviour is typical of molecular machines but is extremely challenging to characterize both from experimental and theoretical viewpoints. In this paper, we present a hybrid experimental/computational approach to address this problem by exploiting the information on molecular shape contained in nuclear magnetic resonance experiments to determine accurate conformation ensembles for the multi-domain enzyme adenylate kinase with help of advanced simulation methods.

## Introduction

Conformational heterogeneity as a consequence of dynamics is an intrinsic feature of proteins linked to biological function.[1] An important aspect for our understanding of protein dynamics is a molecular characterization of the structural states that are populated and of how correlated conformational changes can mediate biological functions such as allostery and signal transduction.[2], [3] The characterization of correlated conformational changes requires a description of how local structural heterogeneity translates into global conformational changes through collective motions. Recent developments in the analysis of various NMR parameters at atomic resolution, such as spin relaxation rates[4] and residual dipolar couplings (RDCs),[5]–[7] have enabled the description of local structural heterogeneity. However, inferences of collective conformational changes from local correlated motions have relied on force fields or motional models.[7]–[9]

**Figure 1:**
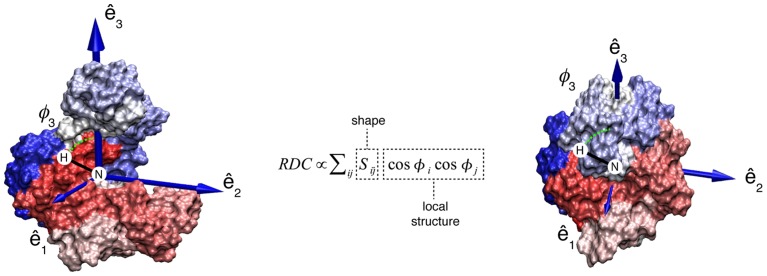
RDCs report on molecular shape. RDCs depend on the geometry of the relevant nuclei (*φ*_i_) and on the degree and direction of alignment (*S*) of the molecule with respect to the laboratory frame. *S* is a 3×3 symmetric and traceless matrix, which contains information on the shape of the molecule. *φ*_i_ is the angle between the inter-nuclear vector and axis i of the molecular reference frame where S is defined. The direction and magnitude of alignment are shown as eigenvectors (ê_i_, i = 1, 2, 3) and eigenvalues (vector size, not in scale) for AK_e_ in the open (left) and closed (right) states.

RDCs are NMR parameters that report on both the local and global structural properties of weakly aligned macromolecules and, under the assumption that alignment does not alter the properties of the protein, can be used to study the amplitude of dynamics, especially when combined with simulations.[5], [10] Alignment can be induced by steric and/or electrostatic interactions of the macromolecule with external media. Specifically, for a given conformation, RDCs depend on the geometrical properties of the environment of the nuclei in the molecular frame and on the direction and degree of alignment[11], [12] (Eq. 1, Fig. 1). 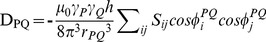
(1)


In Eq. 1, for two nuclei P and Q, *φ_i_^PQ^* is the angle between the inter-nuclear vector and axis *i* of the molecular frame, *S_ij_* is an element of the alignment tensor (*S*), *r_PQ_* is the distance between P and Q, *γ_X_* is the gyromagnetic ratio of nucleus X, *µ_0_* is the magnetic susceptibility of vacuum and *h* is Planck's constant. The alignment tensor *S* is given by the alignment mechanism and, under steric alignment conditions, can be computed accurately and is closely related to the shape of the aligned macromolecule.[12]–[14] RDCs measured in steric alignment combine the local information contained in NMR parameters with the shape information contained in relaxation rates[15]–[17] and small angle X-ray scattering (SAXS).[18] Since inter-domain motions alter the shape of proteins RDCs measured in steric alignment have, as we will show, the potential to act as reporters of inter-domain structural heterogeneity.

RDCs can be used to study the structural heterogeneity of globular and disordered proteins by analysing the effect of (sub-ms) motional averaging on this NMR parameter. The effect of fast (sub-ns) local motions that do not directly alter the magnitude and main directions of alignment can be analysed in the molecular frame defined by the alignment tensor of the average structure.[19], [20] The analysis of motions that change the shape of the protein, that are typically slower than the timescale of alignment (0.5 to 5 ns) [21] needs on the contrary to explicitly consider that the various conformations in fast exchange that contribute to the measured RDC can have different alignment tensors.[20], [22] Here we show that for a molecule undergoing conformational changes in two distal sites the RDCs depend on the degree of correlation of such changes. We exploit this to characterize the degree of correlation of the inter-domain conformational changes occurring in the substrate-free state of *E. coli* Adenylate Kinase (AK_e_), an enzyme that undergoes conformational changes involving shape changes.

## Results and Discussion

Our approach is based on the determination of ensembles that collectively agree with RDCs. The generation of the ensemble is divided in two steps (see Methods and Supporting Information). First, an enumeration (*ca.* 10^5^) of inter-domain orientations is performed using unrestrained simulations. Secondly, the ensemble of minimal size that best fits the RDCs is identified by a genetic algorithm (see Figs. S1 and S2).[23] To maximize the coverage of conformational space in the first step we used PELE, an all atom Monte Carlo simulation algorithm with a move set designed to explore normal modes (see Methods and Supporting Information).[24] The RDCs of each conformer are calculated via Eq. 1 using two independent methods to compute the *S* from knowledge of the structure.[12], [13] In this case we used only the steric tensor because it is related to shape but it is principle possible to use also the electrostatic tensor, particularly when conformational changes modulate the charge distribution.

**Figure 2:**
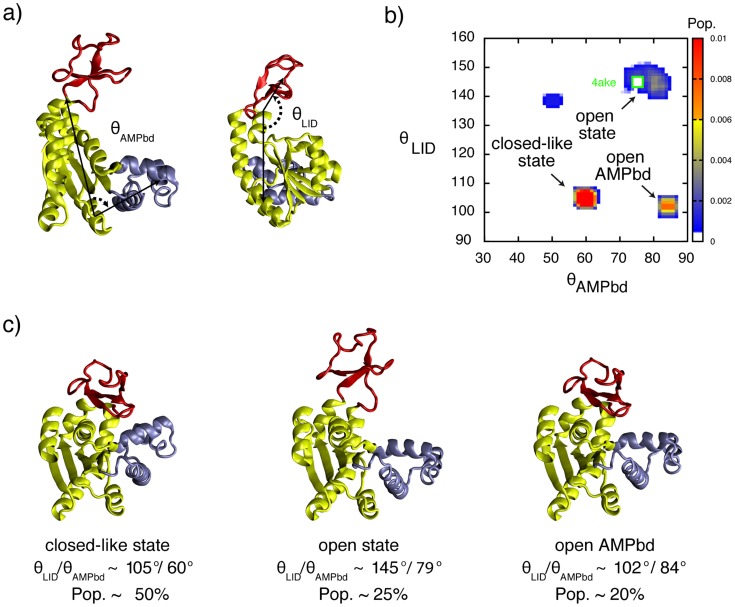
Determination of correlated inter-domain conformational heterogeneity in AK_e_ from RDCs. (A) X-ray structure of substrate free open AK_e_ (pdb code 4ake). The CORE (yellow), LID (red) and AMPbd (cyan) domains are shown. The angles used to describe inter-domain conformational changes (see methods) are shown. (B) Inter-domain orientation distribution for AK_e_. The robustness of the distributions against various sources of error is discussed in the Supporting Information. (C) Representative conformations for each basin are shown together with angles and their estimated populations; little structural heterogeneity occurs within basins. The uncertainties in the populations due to estimated experimental in the measurements of the RDCs and computational errors are estimated to 10%.

AK_e_ is an essential enzyme that catalyses the reversible conversion of ATP and AMP into two ADP molecules. The role of AK_e_ is to maintain the energy balance in the cell, which is essential. AK_e_ is a modular enzyme composed of three sub-domains: a CORE domain responsible for thermal stability[25], [26] and two flexible substrate-binding domains referred to as LID and AMPbd (Fig 2a). Crystallographic structures of AK_e_ have been solved for the open (inactive) and closed (active) states.[27] Substrate-free AK_e_ has been shown to sample a closed-like state using single-molecule Föster resonance energy transfer (smFRET) experiments.[28], [29] We previously reported an analysis of AK_e_ using RDCs in the substrate-free (open) and substrate-bound (closed) states where we fit the RDCs to the corresponding crystallographic structures.[30] We found that the fit of the closed state was better than that of the free state, which we interpreted as evidence for inter-domain dynamics in the substrate-free enzyme.

To identify major conformational states sampled by substrate-free AK_e_ we used backbone NH RDCs measured in steric alignment to select ensembles of conformations from a pool derived from simulations started from the open and closed X-ray structures[27],[31] (see Methods). The ensembles collectively agree with the RDCs (Q≈0.26) even though the structures they contain do not (Q>0.6) when considered individually. An ensemble size of 4 to 64 accounted for the data and equivalent distributions were observed regardless of ensemble size and method used to calculate the RDCs (Figs S3 to S5). It is worth noting that the LID domain of AK_e_ is in equilibrium with a locally unfolded state with a population of *ca.* 5% at 37°C;[32] under the conditions used in this study (25°C) this state has a low population (<1%). Similarly, cracking of the AMPbd along the closing mechanism may play a role.[26], [33] However, cracking occurs at the transition state, and therefore has a very low population. The states obtained for AK_e_ are shown in Fig. 2b. An analysis of the results indicated that substrate free AK_e_ samples three major states corresponding to i) a closed-like state (θ_LID_ ∼105°, θ_AMPbd_ ∼60°, population ∼0.5±0.1), where the LID is closed and the AMPbd slightly opened ii) the open state (θ_LID_ ∼146°, θ_AMPbd_ ∼74°, population ∼0.25±0.1), where both nucleotide binding domains are open, and iii) conformations in which the degree of domain opening is intermediate between that of the fully closed and fully open. The estimate of the fraction closed AK_e_ from our analysis is 0.5, which is in agreement with numbers from single molecule FRET studies where fractions of 0.6[29] and 0.3[28] has been enumerated.

We performed additional control simulations to assess the robustness of the distributions obtained. Scrambling the RDCs lead to a list of restraints that could not be fit to any distribution (Fig. S6), showing that the distribution obtained is encoded in the data and not biased by the population of the conformers in the pool. The distribution shown in Fig. 2a was found to be robust to various sources of error, including errors in the experimental RDCs (Fig. S7) and in the prediction of the alignment tensor (Fig. S8). We also performed computational experiments to ensure that a single set of RDCs suffices to distinguish between ensembles with a distinct number of states and identify correlated structural changes (see Figs. S9 to S13).

An analysis of the conformational ensemble allowed us to assess the presence and degree of inter-domain correlation, an important property of AK_e_. As shown in Fig. 2b, the ATP and AMP domain movements are correlated, disfavouring conformations where the LID is open and the AMPbd is closed. Our findings agree with free energy calculations[34], molecular dynamics simulations[35] as well as with normal mode analysis.[33], [36] Although it is impossible to derive the closure pathway from equilibrium data, the conformational states observed favour a step-wise mechanism in which LID closure takes place before AMPbd closure. Such a mechanism would be beneficial because the initial closure of the LID, together with the substantially higher affinity of this domain for its substrate (ATP) as compared to AMP (50 *vs* 1700 µM),[30] would reduce the probability of non-productive binding of AMP to the LID.[37]

Single-molecule FRET[28], [29] and NMR[28] have shown that the open and closed states of AK_e_ are in equilibrium and that nucleotide binding shifts it towards the closed state.[30] Of particular interest are smFRET experiments, as these can resolve conformational states and provide a qualitative validation of the ensemble. Two different reaction coordinates, corresponding to distances between residues in the LID and the AMPbd (*Aquifex* AK)[28] and between residues in the LID and the CORE domain (AK_e_),[29] have been studied. In the former the authors resolved two states in which open conformations were populated to *ca.* 70%, whereas in the latter the authors found that the open conformation was disfavoured, with a population of *ca.* 40%. These results can be rationalized based on the ensemble. Following the approach of Beckstein *et al.*,[38] where distances corresponding to the open and closed states are mapped in domain angle space, we estimated open populations along the LID-AMPbd and LID-CORE smFRET reaction coordinates >50% and ∼30%, respectively (for LID-AMPbd large uncertainties are found due to difficulties in assigning open and closed states as quantified by smFRET). Overall, the ensemble is able to qualitatively reconcile two apparently contradictory smFRET studies, which also suggests that steric alignment does not significantly alter the structural heterogeneity of AK_e_.

Here we have used the dependence of RDCs on global molecular shape *via S* to determine ensembles reporting on the amplitude and degree of correlation of inter-domain motions. Although RDCs are uniquely suited for this, this is a challenging endeavour due to their intrinsic degeneracies.[39] It is therefore relevant to discuss the factors that allowed the approach used in this work to alleviate them. The first factor is that our approach accounts for how shape changes alter the value of the RDC by computing *S* for each inter-domain orientation *i.e.* two inter-domain orientations that would have degenerate RDCs when the tensor is assumed to be constant can be differentiated if they have sufficiently large differences in shape (Fig. S14).[39] A second factor is the presence of structural constraints due to the covalent linkage, where steric clashes between domains restrict the available conformational space;[40], [41] AK_e_ is a well-structured proteins where this effect is particularly important but for multi-domain proteins with flexible linker sequences RDCs measured in multiple alignment media will be required. Alternatively, RDCs could be complemented with structural restraints derived from smFRET or SAXS data.

Correlated motions in proteins are thought to mediate biochemical functionality.[42] Recently, we have identified weak long-range correlated motions in a surface patch of ubiquitin involved in molecular recognition.[7] Here, we move a step forward and find correlated domain movements in a representative multi-domain enzyme. The observed inter-domain correlation suggests functional roles in allowing ligand access, in adopting the inter-domain orientation necessary for catalysis as well as in binding allostery. Our results, therefore, reinforce that correlated inter-domain motions in proteins can mediate important biochemical processes.

## Methods

### RDC calculation

NH RDCs used in this work for free AK_e_ were measured in stretched polyacrylamide gels.[30] Two independent methods, PALES [12] and ALMOND[13], [13]were used to calculate the alignment tensor using Eq. 1. They gave equivalent distributions as shown in Figs. S3–S5. In Figs. 2 we provide the distributions that lead the best agreement between calculated and experimental RDCs [43] (Tab. S1). For AK_e_ it was obtained using ALMOND. The agreement between calculated and experimental (or reference) RDCs was assessed by the quality factor Q [43] defined in Eq. 2. 
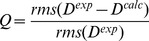
(2)


The calculated RDCs were scaled, after averaging across the ensemble, to minimize Q to account for the difficulty of predicting the absolute concentration of alignment medium. We used RDCs corresponding to structured regions in the protein (see Fig. S15). For AKe, we found that RDCs in loop regions were more difficult to fit and therefore we decided to exclude them. However, we note that the ensemble derived including RDCs in the loop regions remains unaffected. We also evaluated the impact of the alignment media by monitoring changes in chemical shifts induced by the presence of the stretched polyacrylamide gel. As shown in the Supporting Figure S16, no significant chemical shift perturbations were observed when apo AK_e_ was immersed into the anisotropic phase. As a reference the chemical shift perturbations induced by binding of the inhibitor Ap5A to AK_e_ are also displayed. A thorough analysis of the robustness of the procedure to various source of error is provided as Supporting Information. To select the optimum ensemble size we monitored the agreement with RDCs used to guide the genetic algorithm and to RDCs left out of this process or free RDCs. Ten sets of randomly removed RDCs (20%) or free RDCs sets were used and 20 independent calculations were run for each set of randomly removed RDCs. The ensemble was qualitatively validated against two independent smFRET experiments (see results and discussion).

### Reference pool of conformations

An extensive set of inter-domain conformations (*ca.* 10^5^) was generated for AK_e_. The X-ray structures 1ake [31] and 4ake [27] were used as seeds. Trajectories were generated where either the AMP or ATP binding domain open (or closed). From each state along the trajectory a second trajectory was generated where the other domain was forced to open (or to close). These simulations were performed with the molecular simulation package CHARMM c35.[44] A time step of 1.0 fs was used. Simulations were performed at 300K. A shape-term (biasing) potential on the backbone atoms was used. The CHARMM commands "CONStraint HARmonic BEStfit COMParison" and the IC table tool (CONStraint IC BOND ANGL IMPR DIHE) were used to sample inter-domain orientations between open and closed conformations. The initial force was set to 0.1 and exponentially increased by a factor of 1.05 during 200 cycles of 100 steps each. The degree of opening ranged between ∼30–90° and ∼90–160° for the AMP- and ATP-binding domains (see angle definitions for AK_e_). The angles sampled covered the range observed by known X-ray structures of AK_e_ and related proteins. [38] An advanced sampling strategy based on the PELE [24] method was used (see Protein Energy Landscape Exploration, see Supporting Information). This allowed identifying high-energy conformations which are unlikely to be of sufficiently low energy to be present in the crystalline state or sampled by conventional MD.

### Searching algorithm

An in-house genetic algorithm GA was developed to efficiently search within the pool of structures used to determine the experimental inter-domain orientation distributions of T4L and AK_e_. Initially 1000 ensembles were generated with structures randomly selected from a pool of ca. 10^5^ conformations (see reference pool of conformations above). Ensembles of size N = 1, 2, 4, 8, 16, 32 and 64 were used. The 10^3^ ensembles of size N were submitted to evolution through 1000 steps. At each step two new sets of 10^3^ ensembles were generated by mutation and crossing operations, leaving 3×10^3^ ensembles. At each step the best 10^3^ ensembles, based on the value of Q (see below), were retained. After 1000 iterations the best ensemble was saved and the complete procedure was repeated 200 times. Because too high mutation and recombination rates may lead to loss of good solutions and to premature convergence of the GA, these parameters evolved during the calculations. Initially, the mutation and crossing rates were set to 100% and 2%, respectively. At each step new mutation and crossing rates were determined by dividing/multiplying them by a factor of 1.001, respectively. In Figs. S1 and S2 a scheme illustrating the GA and the convergence of the method are shown. The error in the population of the states observed were determined from the influence of RDC experimental uncertainty by performing 200 calculations with random Gaussian error added to each experimental RDC. We used a standard deviation of 1.0 Hz for AK_e_, three times the estimated experimental error.

### Inter-domain angle definitions

The definition used by Beckstein *et al.* [38] was adopted in this work. Briefly, the angle formed between the AMP-binding domain (AMPbd) and the core domain (CORE) was determined as the angle formed by two vectors: Vector 1 connects the centers of mass (Cα atoms) of CORE residues 90–100 with residues 115–125 (“hinge region” between the CORE and LID domains). Vector 2 connects the centers of mass of CORE residues 90–100 and AMPbd residues 35–55. The angle formed between the ATP-binding domain (LID) and the CORE was defined equivalently using the angle formed between two vectors: Vector 1 connects the centers of mass of residues 115–125 (“hinge region” CORE–LID domains) with CORE residues 179–185. Vector 2 connects the centers of mass for residues 115–125 (“hinge region” CORE–LID) with LID residues 125–153.

## Supporting Information

Figure S1 **Schematic flow-chart showing the steps involved in the calculation of inter-domain conformational heterogeneity from RDCs.** In step 1 a pool of conformations is generated. In step 2 an algorithm for searching ensembles of structures that match experimental data is used (see Methods in the main text). The algorithm randomly selects 10^3^ ensembles of size N from a pool of *ca.* 10^5^ conformations generated in the first step. For each value of N the agreement of the ensemble with the experimental RDCs is optimized by substituting ensemble members with structures of the pool as well as by swapping structures between ensembles. The best 10^3^ ensembles generated in the second step are retained and the process is repeated until agreement between calculated and experimental RDCs is reached. The optimal value of N is determined by monitoring the agreement with RDCs used to guide the genetic algorithm (Q_work_) and RDCs left out of the calculation (free RDCs, Q_free_).
(TIF)

Figure S2 **Genetic algorithm.** **a**, Convergence of the algorithm developed in this work to select ensembles of structures that match RDCs. Both data sets, restrained (Q_work_) and unrestrained RDCs (Q_free_), reached a plateau region after 400 iterations. **b**, Mutation and crossing operation rates applied at each step of the ensemble refinement.
(TIF)

Figure S3 **Fitting of NH RDCs for AK_e_ and determination of the optimum ensemble size by monitoring the agreement with RDCs used to guide the genetic algorithm and RDCs left out of the calculation (20%).** The results obtained using two independent methods to calculate the alignment tensor, ALMOND and PALES, are shown. The Q_factor_ for working (Q_work_; restrained) and free (Q_free_; unrestrained) RDCs are shown (see Supporting Text S1). For each ensemble size 200 independent calculations were performed and the results pooled.
(TIF)

Figure S4 **Experimental AK_e_ inter-domain orientation distributions obtained for ensembles of several sizes (see Supporting Text S1).** The distributions were obtained using the ALMOND method to calculate the alignment tensor (see Fig. S5 for distributions obtained using PALES). For each ensemble size 200 independent calculations were performed and the results pooled. The x and y axis correspond to the θ_LID_ and θ_AMPbd_ angles (in degrees) shown in Fig. 3a.
(TIF)

Figure S5 **Experimental AK_e_ inter-domain orientation distributions obtained for ensembles of several sizes (see Supporting Text S1).** The distributions were obtained using PALES to calculate the alignment tensor (see Fig. S4 for distributions obtained using the ALMOND method). For each ensemble size 200 independent calculations were performed and the results pooled. The x and y axis correspond to the θ_AMPbd_ and θ_LID_ angles (in degrees) shown in Fig. 2a.
(TIF)

Figure S6 **Distributions obtained for AK_e_ after randomly scrambling the experimental RDCs to obtain incorrect lists of restraints (see Supporting Text S1).** The Q factor obtained in all cases was >0.9, indicating that it was not possible to fit the incorrect data to any physically possible distribution of inter-domain orientations; for each ensemble size a different list of randomly scrambled RDCs was used. For AK_e_, the x and y axis correspond to the θ_AMPbd_ and θ_LID_ angles (in degrees) shown in Fig. 2a.
(TIF)

Figure S7 **Impact of error in the experimentally measured RDCs of AK_e_.** The fitting of RDCs (Q_work_) as well as the distributions obtained are shown. Random Gaussian error was added to the RDCs prior to ensemble calculations. The results shown were obtained using the ALMOND method to calculate the alignment tensor (see Supporting Text S1). For AK_e_, The x and y axis correspond to the θ_AMPbd_ and θ_LID_ angles (in degrees) shown in Fig. 2a.
(TIF)

Figure S8 **Experimental distributions of AK_e_ as a function of error in the prediction of the alignment magnitude (see Supporting Text S1).** The RDCs of each conformation were scaled by a number which depended on its position along the RC(s) used in this work. The error was increased exponentially along the RC(s) reaching at the end points of the RC a value of 2, 3, 10, 20 and 50 fold. The x and y axis correspond to the θ_AMPbd_ and θ_LID_ angles (in degrees) shown in Fig. 2a.
(TIF)

Figure S9 **Reconstruction of a synthetic (computer designed) unimodal distribution represented by a magenta box with an increasing number of conformations, N = 1, 8 and 32.** Ensembles with 8 or more members fit equally well the synthetic RDCs and best predicted RDCs (20%) left out of the calculations (see Supporting Text S1). The x and y axis correspond to the θ_AMPbd_ and θ_LID_ angles (in degrees) shown in Fig. 2a.
(TIF)

Figure S10 **Reconstruction of a synthetic (computer designed) four state distribution represented by magenta boxes of equal population with N = 1, 2, 4, 8, 16 and 32.** Ensembles with 16 or more members fit equally well the synthetic RDCs and best predicted RDCs (20%) left out of the calculations (see Supporting Text S1). The x and y axis correspond to the θ_AMPbd_ and θ_LID_ angles (in degrees) shown in Fig. 2a.
(TIF)

Figure S11 **Reconstruction of synthetic unimodal inter-domain orientation distributions of AK_e_ represented by a magenta box from NH RDCs (see Supporting Text S1).** Distributions rebuilt with added random Gaussian are shown (standard deviations of 1%, 3% and 6% with respect to the maximum coupling). The best ensemble size was determined by monitoring the agreement with RDCs used to guide the genetic algorithm (Q_work_) and RDCs left out of the calculation (free RDCS, Q_free_) (**Fig. S13**). The x and y axis correspond to the θ_AMPbd_ and θ_LID_ angles (in degrees) shown in Fig. 2a.
(TIF)

Figure S12 **Reconstruction of synthetic multi-modal inter-domain orientation distributions of AK_e_ represented by magenta boxes of equal population from NH RDCs (see Supporting Text S1).** Distributions rebuilt with added random Gaussian error with standard deviations corresponding to 1%, 3% and 6% of error respect the maximum coupling are shown. The best ensemble size was determined by monitoring the agreement with RDCs used to guide the genetic algorithm (Q_work_) and RDCs left out of the calculation (free RDCS, Q_free_) (Fig. S13). The x and y axis correspond to the θ_AMPbd_ and θ_LID_ angles (in degrees) shown in Fig. 2a.
(TIF)

Figure S13 **Agreement with RDCs left out of the calculations (20%) for AK_e_ protein for the synthetic distributions shown in Figs. S11 and S12 (see Supporting Text S1).** The Q_factor_ for working (Q_work_; restrained) and free (Q_free_; unrestrained) RDCs is shown. Distributions rebuilt with added random Gaussian error with standard deviations of 1% (first row), 3% (second row) and 6% (third row) of error with respect the maximum coupling are shown.
(TIF)

Figure S14 **Maximum deviation in the fitted populations as a function of the alignment tensor prediction error for AK_e_ protein for computer designed distributions (see Supporting Text S1).** The target distributions covered closed/open states whose populations varied between 0% to 100% in steps of 1 percent. For each degree of random error added 100 independent runs were performed.
(TIF)

Figure S15 **Sequence (first raw) and secondary structure of AKe in the open (pdb code 4ake; second raw) and closed (pdb code 1ake; third raw) states.** $ indicates that the RDC was used in the calculations. Residues labeled with * were excluded. The label – indicates that the RDC was not available. The AMPbd (residues 28–72) and LID (113–176) domains are highlighted in red and yellow, respectively. Residues 1–27, 73–112 and 176–214 correspond to the CORE domain.
(TIF)

Figure S16 **Alignment of AK_e_ does not induce significant chemical shift perturbations.** Shown are chemical shift perturbations to apo AK_e_ induced by the inhibitor Ap5A (red) and by introduction of the enzyme into the anisotropic stretched polyacrylamide gel (black). Chemical shift perturbations were calculated according to: δω =  0.2|Δ^15^N|+| Δ^1^H |.
(TIF)

Table S1 **Agreement between the calculated and experimental RDCs for AK_e_.** *S* ^calc^ refers to the result obtained when *S* is predicted based on the atomic coordinates of each protein conformation, whereas *S* ^fit^ refers to the result obtained when *S* was fit using single value decomposition. The Q values are those of the final ensemble.
(DOCX)

Table S2 **Average structural root-mean-squared deviation (RMSD) between the calculated ensemble members and reference X-ray structures for AK_e_.**
(DOCX)

Table S3 **NH RDCs measured for E. coli AK_e_.** RDCs used in ensemble refinement are shown in Fig. S15 (average experimental error <0.3 Hz).
(DOCX)

Text S1 **Supporting methods.**
(DOCX)
