# Supplementary figures and images for: Correlated Inter-Domain Motions in Adenylate Kinase
Source: PLoS Comput Biol. 2014 Jul 31;10(7):e1003721. doi: 10.1371/journal.pcbi.1003721 (PMC4117416; doi:10.1371/journal.pcbi.1003721)

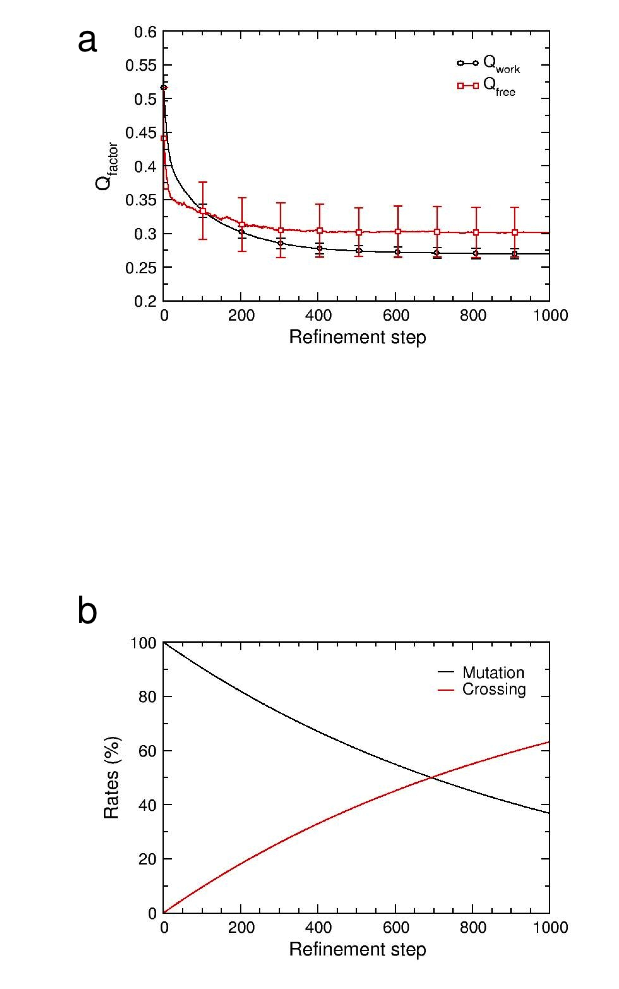

Supplement: Figure S2 — Genetic algorithm. a, Convergence of the algorithm developed in this work to select ensembles of structures that match RDCs. Both data sets, restrained (Qwork) and unrestrained RDCs (Qfree), reached a plateau region after 400 iterations. b, Mutation and crossing operation rates applied at each step of the ensemble refinement. (TIF) [file pcbi.1003721.s002.tif]

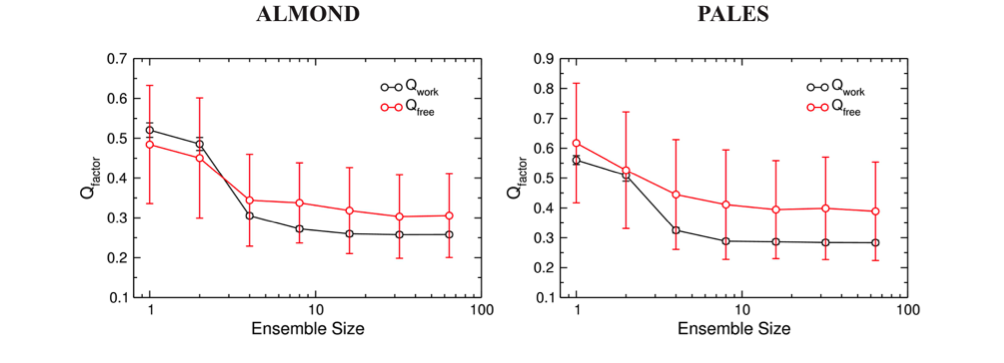

Supplement: Figure S3 — Fitting of NH RDCs for AKe and determination of the optimum ensemble size by monitoring the agreement with RDCs used to guide the genetic algorithm and RDCs left out of the calculation (20%). The results obtained using two independent methods to calculate the alignment tensor, ALMOND and PALES, are shown. The Qfactor for working (Qwork; restrained) and free (Qfree; unrestrained) RDCs are shown (see Supporting Text S1). For each ensemble size 200 independent calculations were performed and the results pooled. (TIF) [file pcbi.1003721.s003.tif]

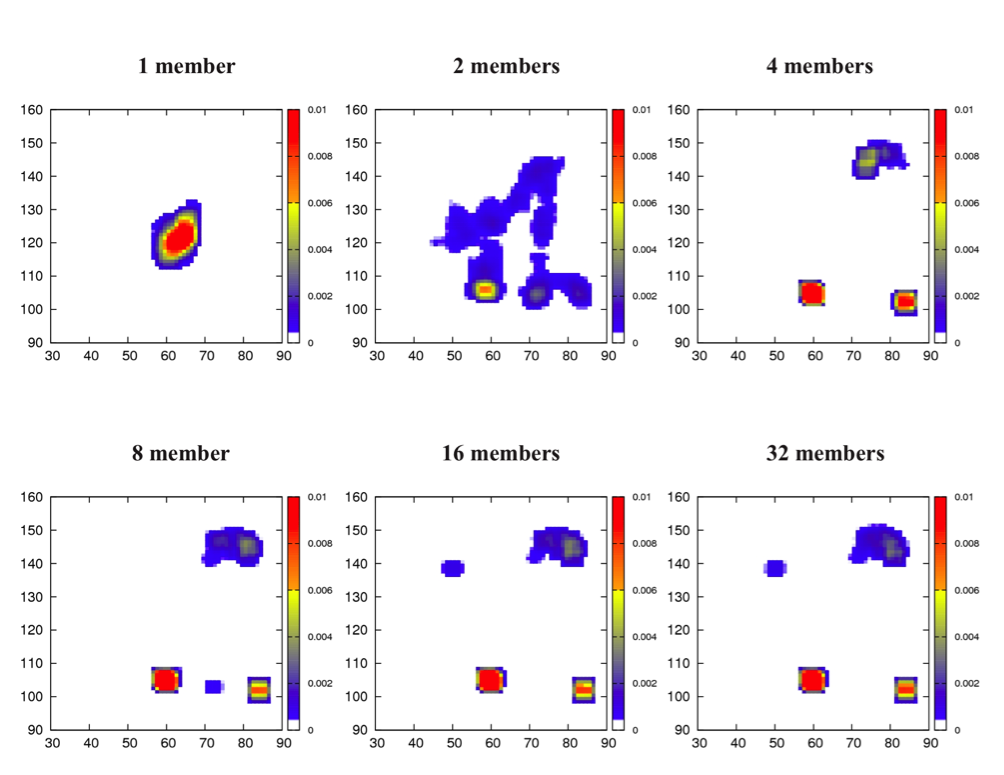

Supplement: Figure S4 — Experimental AKe inter-domain orientation distributions obtained for ensembles of several sizes (see Supporting Text S1). The distributions were obtained using the ALMOND method to calculate the alignment tensor (see Fig. S5 for distributions obtained using PALES). For each ensemble size 200 independent calculations were performed and the results pooled. The x and y axis correspond to the θLID and θAMPbd angles (in degrees) shown in Fig. 3a. (TIF) [file pcbi.1003721.s004.tif]

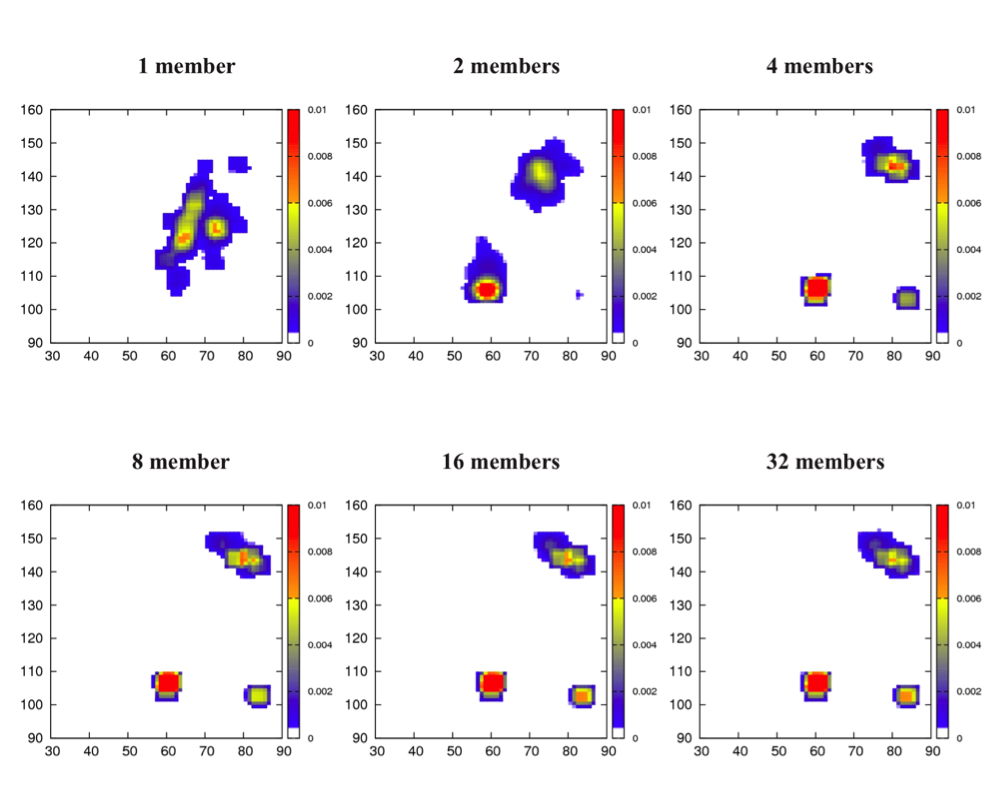

Supplement: Figure S5 — Experimental AKe inter-domain orientation distributions obtained for ensembles of several sizes (see Supporting Text S1). The distributions were obtained using PALES to calculate the alignment tensor (see Fig. S4 for distributions obtained using the ALMOND method). For each ensemble size 200 independent calculations were performed and the results pooled. The x and y axis correspond to the θAMPbd and θLID angles (in degrees) shown in Fig. 2a. (TIF) [file pcbi.1003721.s005.tif]

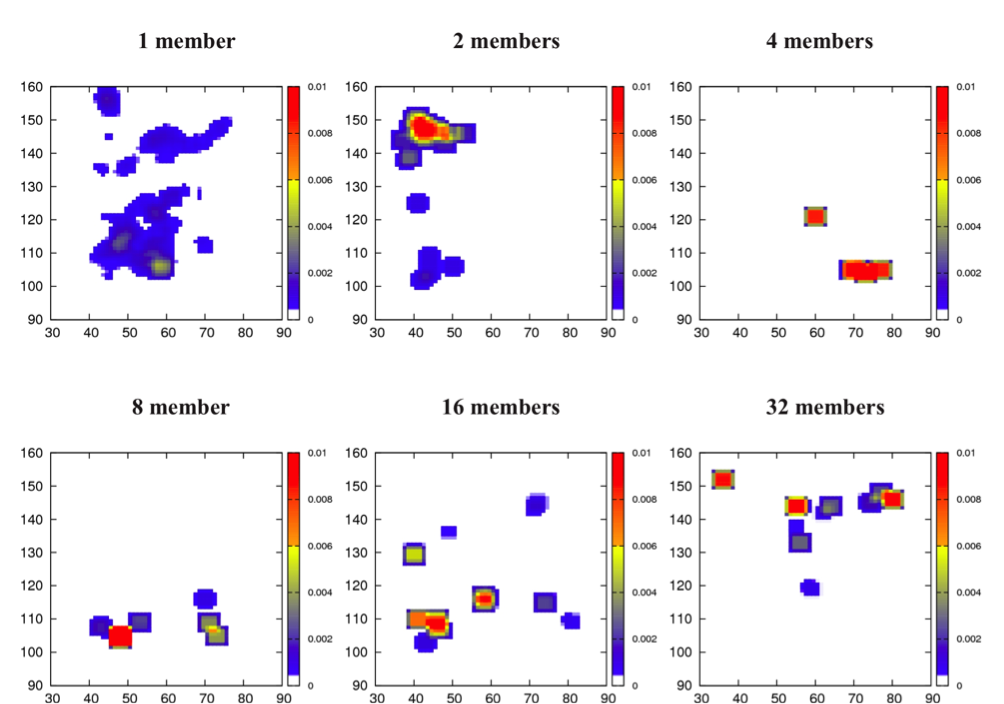

Supplement: Figure S6 — Distributions obtained for AKe after randomly scrambling the experimental RDCs to obtain incorrect lists of restraints (see Supporting Text S1). The Q factor obtained in all cases was >0.9, indicating that it was not possible to fit the incorrect data to any physically possible distribution of inter-domain orientations; for each ensemble size a different list of randomly scrambled RDCs was used. For AKe, the x and y axis correspond to the θAMPbd and θLID angles (in degrees) shown in Fig. 2a. (TIF) [file pcbi.1003721.s006.tif]

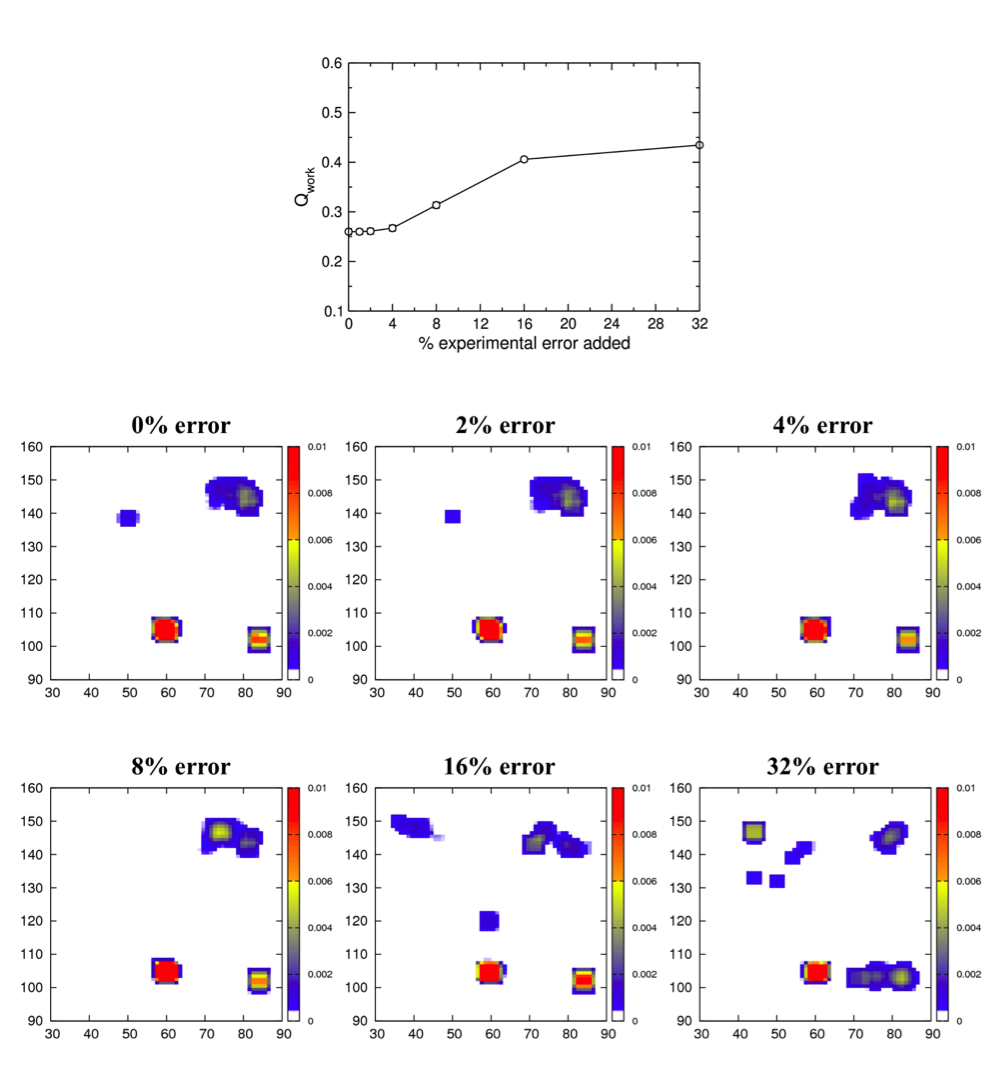

Supplement: Figure S7 — Impact of error in the experimentally measured RDCs of AKe. The fitting of RDCs (Qwork) as well as the distributions obtained are shown. Random Gaussian error was added to the RDCs prior to ensemble calculations. The results shown were obtained using the ALMOND method to calculate the alignment tensor (see Supporting Text S1). For AKe, The x and y axis correspond to the θAMPbd and θLID angles (in degrees) shown in Fig. 2a. (TIF) [file pcbi.1003721.s007.tif]

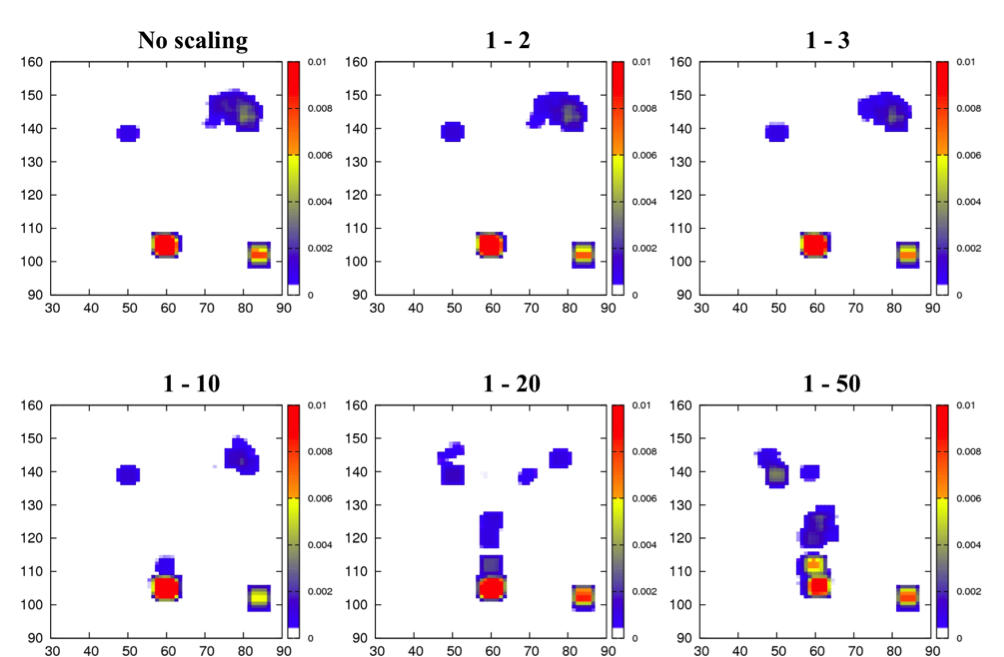

Supplement: Figure S8 — Experimental distributions of AKe as a function of error in the prediction of the alignment magnitude (see Supporting Text S1). The RDCs of each conformation were scaled by a number which depended on its position along the RC(s) used in this work. The error was increased exponentially along the RC(s) reaching at the end points of the RC a value of 2, 3, 10, 20 and 50 fold. The x and y axis correspond to the θAMPbd and θLID angles (in degrees) shown in Fig. 2a. (TIF) [file pcbi.1003721.s008.tif]

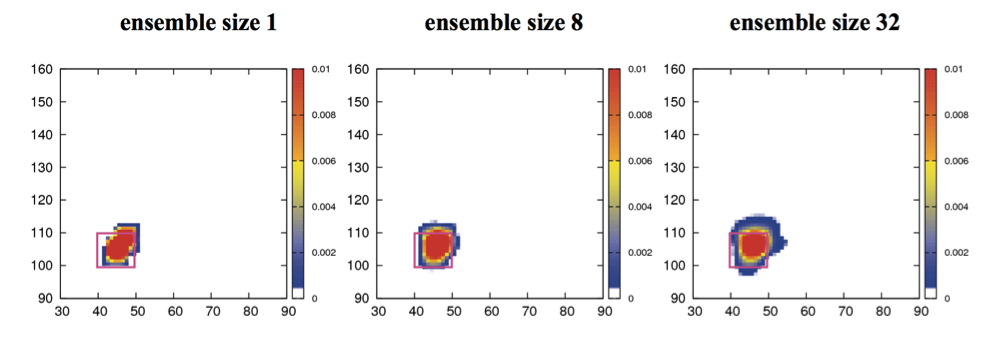

Supplement: Figure S9 — Reconstruction of a synthetic (computer designed) unimodal distribution represented by a magenta box with an increasing number of conformations, N = 1, 8 and 32. Ensembles with 8 or more members fit equally well the synthetic RDCs and best predicted RDCs (20%) left out of the calculations (see Supporting Text S1). The x and y axis correspond to the θAMPbd and θLID angles (in degrees) shown in Fig. 2a. (TIF) [file pcbi.1003721.s009.tif]

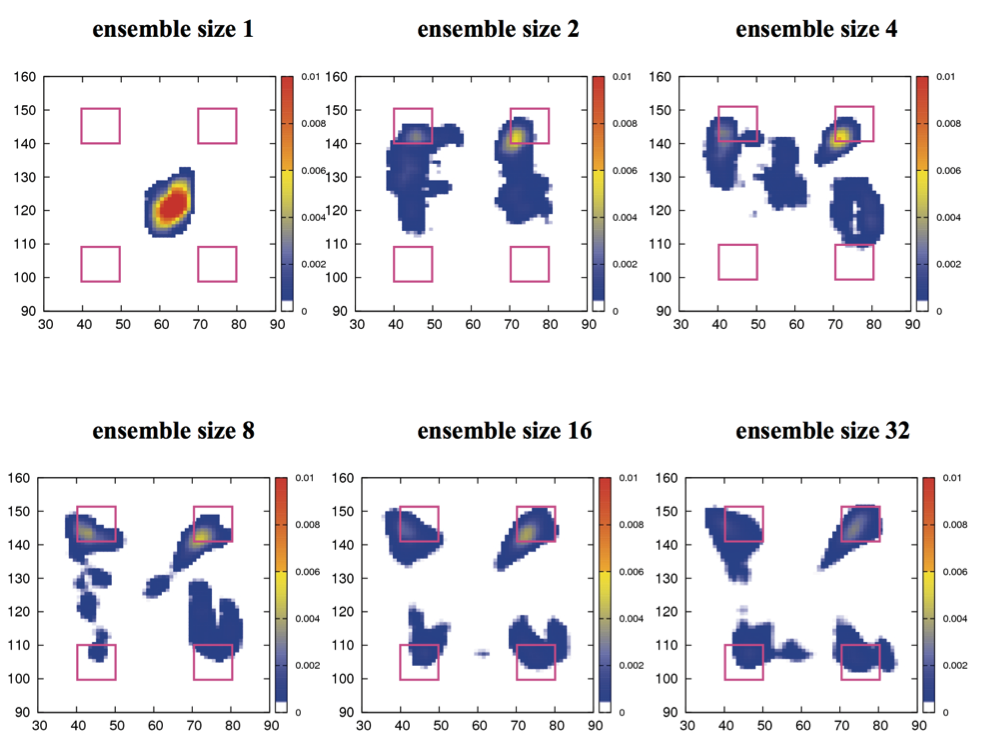

Supplement: Figure S10 — Reconstruction of a synthetic (computer designed) four state distribution represented by magenta boxes of equal population with N = 1, 2, 4, 8, 16 and 32. Ensembles with 16 or more members fit equally well the synthetic RDCs and best predicted RDCs (20%) left out of the calculations (see Supporting Text S1). The x and y axis correspond to the θAMPbd and θLID angles (in degrees) shown in Fig. 2a. (TIF) [file pcbi.1003721.s010.tif]

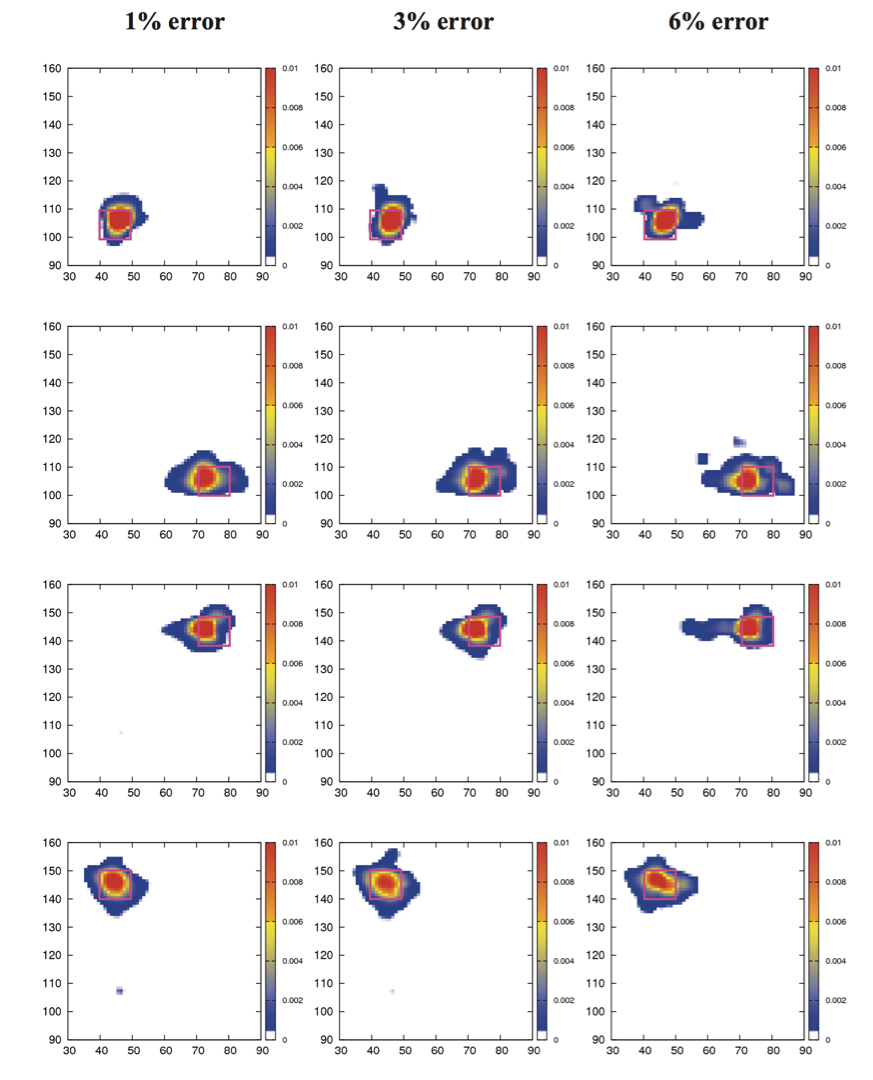

Supplement: Figure S11 — Reconstruction of synthetic unimodal inter-domain orientation distributions of AKe represented by a magenta box from NH RDCs (see Supporting Text S1). Distributions rebuilt with added random Gaussian are shown (standard deviations of 1%, 3% and 6% with respect to the maximum coupling). The best ensemble size was determined by monitoring the agreement with RDCs used to guide the genetic algorithm (Qwork) and RDCs left out of the calculation (free RDCS, Qfree) (Fig. S13). The x and y axis correspond to the θAMPbd and θLID angles (in degrees) shown in Fig. 2a. (TIF) [file pcbi.1003721.s011.tif]

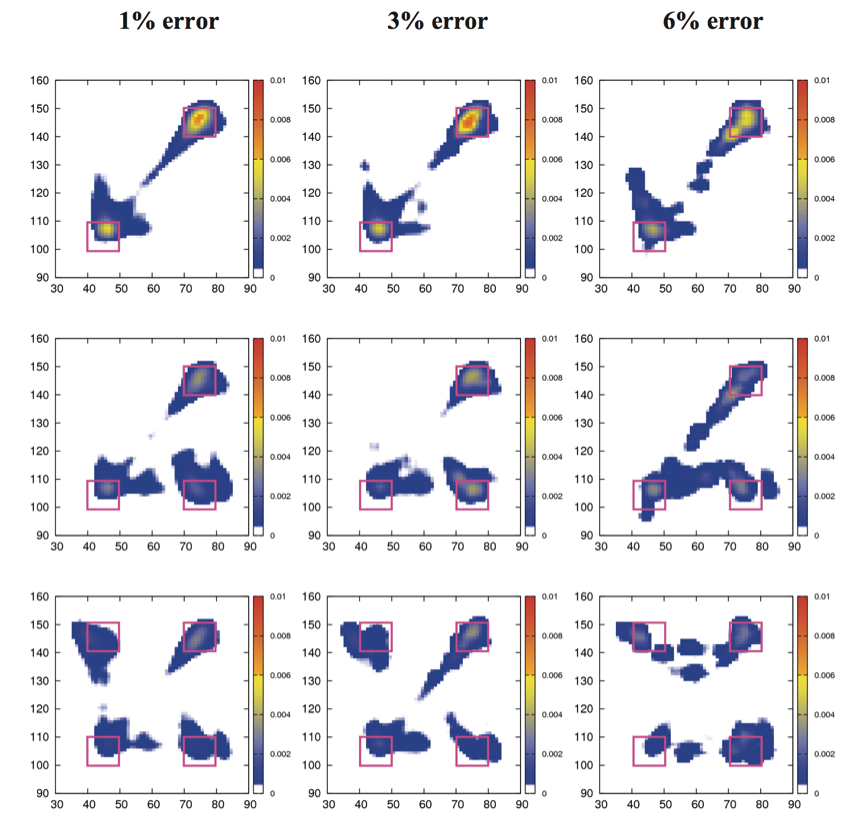

Supplement: Figure S12 — Reconstruction of synthetic multi-modal inter-domain orientation distributions of AKe represented by magenta boxes of equal population from NH RDCs (see Supporting Text S1). Distributions rebuilt with added random Gaussian error with standard deviations corresponding to 1%, 3% and 6% of error respect the maximum coupling are shown. The best ensemble size was determined by monitoring the agreement with RDCs used to guide the genetic algorithm (Qwork) and RDCs left out of the calculation (free RDCS, Qfree) (Fig. S13). The x and y axis correspond to the θAMPbd and θLID angles (in degrees) shown in Fig. 2a. (TIF) [file pcbi.1003721.s012.tif]

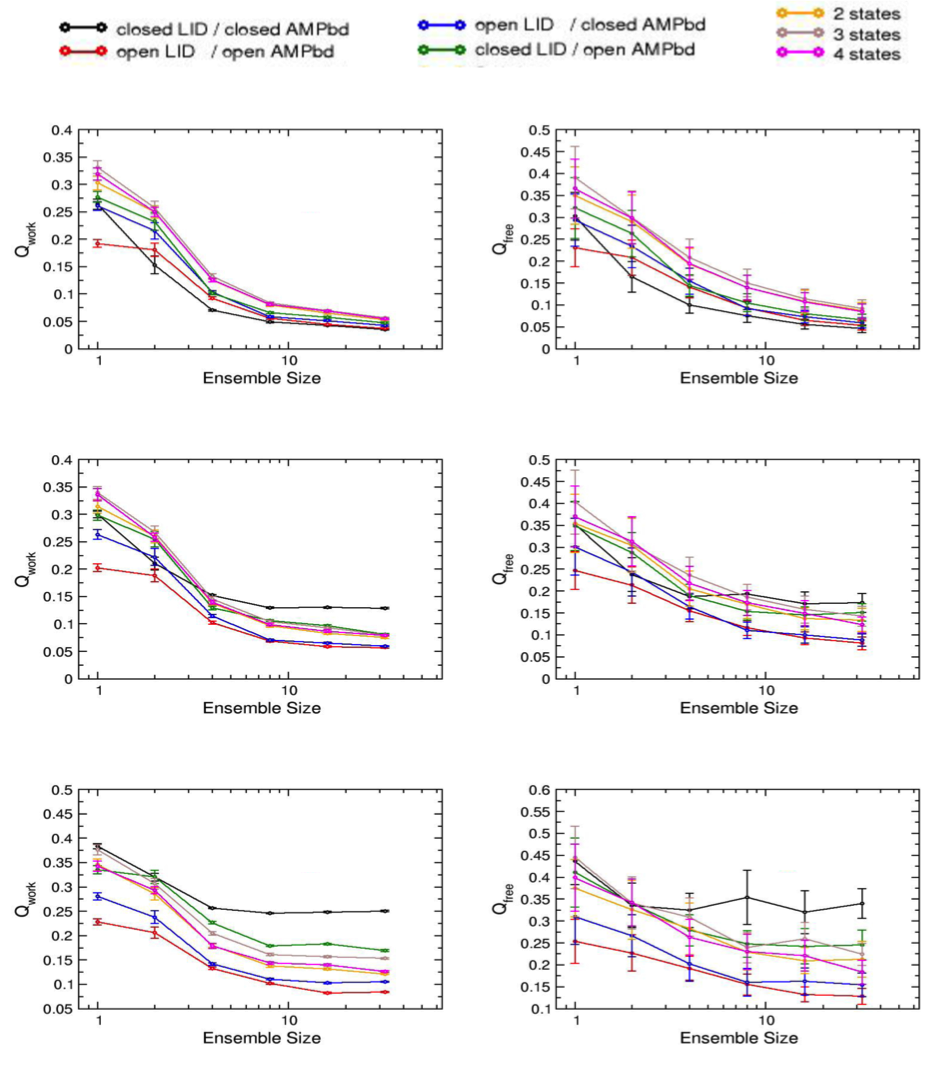

Supplement: Figure S13 — Agreement with RDCs left out of the calculations (20%) for AKe protein for the synthetic distributions shown in Figs. S11 and S12 (see Supporting Text S1). The Qfactor for working (Qwork; restrained) and free (Qfree; unrestrained) RDCs is shown. Distributions rebuilt with added random Gaussian error with standard deviations of 1% (first row), 3% (second row) and 6% (third row) of error with respect the maximum coupling are shown. (TIF) [file pcbi.1003721.s013.tif]

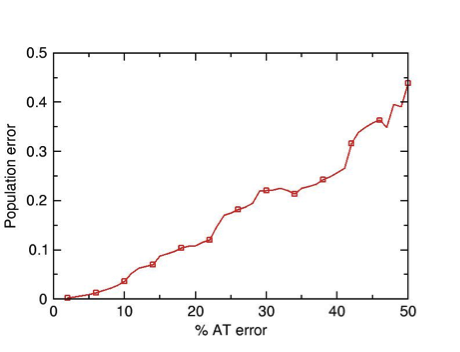

Supplement: Figure S14 — Maximum deviation in the fitted populations as a function of the alignment tensor prediction error for AKe protein for computer designed distributions (see Supporting Text S1). The target distributions covered closed/open states whose populations varied between 0% to 100% in steps of 1 percent. For each degree of random error added 100 independent runs were performed. (TIF) [file pcbi.1003721.s014.tif]

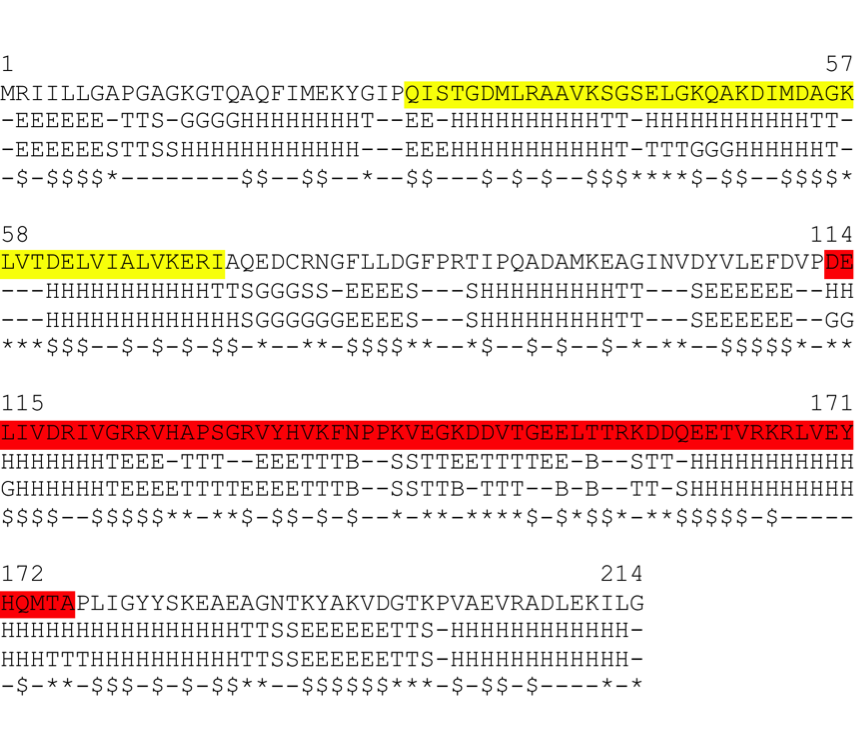

Supplement: Figure S15 — Sequence (first raw) and secondary structure of AKe in the open (pdb code 4ake; second raw) and closed (pdb code 1ake; third raw) states. $ indicates that the RDC was used in the calculations. Residues labeled with * were excluded. The label – indicates that the RDC was not available. The AMPbd (residues 28–72) and LID (113–176) domains are highlighted in red and yellow, respectively. Residues 1–27, 73–112 and 176–214 correspond to the CORE domain. (TIF) [file pcbi.1003721.s015.tif]

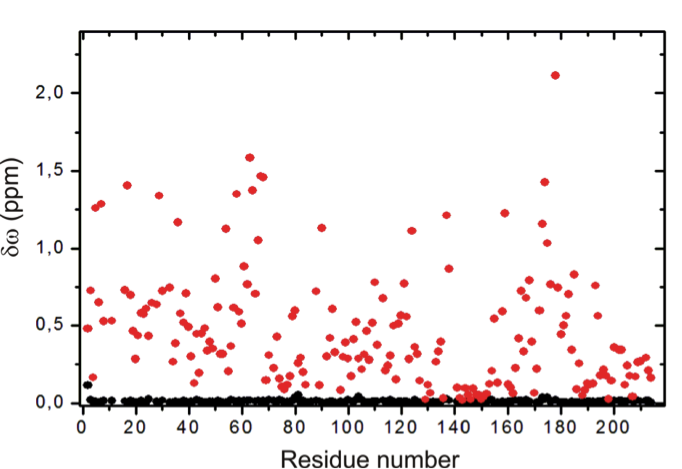

Supplement: Figure S16 — Alignment of AKe does not induce significant chemical shift perturbations. Shown are chemical shift perturbations to apo AKe induced by the inhibitor Ap5A (red) and by introduction of the enzyme into the anisotropic stretched polyacrylamide gel (black). Chemical shift perturbations were calculated according to: δω = 0.2|Δ15N|+| Δ1H |. (TIF) [file pcbi.1003721.s016.tif]
