# Supplementary material for: Correlated Inter-Domain Motions in Adenylate Kinase
Source: PLoS Comput Biol. 2014 Jul 31;10(7):e1003721. doi: 10.1371/journal.pcbi.1003721 (PMC4117416; doi:10.1371/journal.pcbi.1003721)
Supplement: Table S1 — Agreement between the calculated and experimental RDCs for AKe. S calc refers to the result obtained when S is predicted based on the atomic coordinates of each protein conformation, whereas S fit refers to the result obtained when S was fit using single value decomposition. The Q values are those of the final ensemble. (DOCX) [file pcbi.1003721.s017.docx]

| **Q** |  | **S^calc^** | **S^fit^** |
| --- | --- | --- | --- |
| **AK_e_** | **ALL** | 0.26 | *n.a.* |
|  | **CORE** | 0.31 | 0.23 |
|  | **LID** | 0.26 | 0.33 |
|  | **AMPbd** | 0.12 | 0.06 |
