# Supplementary material for: Correlated Inter-Domain Motions in Adenylate Kinase
Source: PLoS Comput Biol. 2014 Jul 31;10(7):e1003721. doi: 10.1371/journal.pcbi.1003721 (PMC4117416; doi:10.1371/journal.pcbi.1003721)
Supplement: Table S2 — Average structural root-mean-squared deviation (RMSD) between the calculated ensemble members and reference X-ray structures for AKe. (DOCX) [file pcbi.1003721.s018.docx]

|  |  | **Ensemble vs**  **1ake X-ray structure** | **Ensemble vs**  **4ake X-ray structure** | **1ake vs 4ake** |
| --- | --- | --- | --- | --- |
| **AK_e_** | **CORE** | 1.35 Å | 1.02 Å | 1.19 Å |
|  | **LID** | 0.98 Å | 2.25 Å | 2.69 Å |
|  | **AMPbd** | 1.89 Å | 0.93 Å | 1.97 Å |
