# Supplementary material for: Correlated Inter-Domain Motions in Adenylate Kinase
Source: PLoS Comput Biol. 2014 Jul 31;10(7):e1003721. doi: 10.1371/journal.pcbi.1003721 (PMC4117416; doi:10.1371/journal.pcbi.1003721)
Supplement: Table S3 — NH RDCs measured for E. coli AKe. RDCs used in ensemble refinement are shown in Fig. S15 (average experimental error <0.3 Hz). (DOCX) [file pcbi.1003721.s019.docx]

| **Res** | **Nuc** | **Res** | **Nuc** | **D/Hz** | - - 1. **Error/Hz** |
| --- | --- | --- | --- | --- | --- |
| 2 | N | 2 | H | −1.399 | - - 1. 0.310 |
| 4 | N | 4 | H | −0.182 | - - 1. 0.460 |
| 5 | N | 5 | H | −1.825 | - - 1. 0.317 |
| 6 | N | 6 | H | −2.555 | - - 1. 0.442 |
| 8 | N | 8 | H | −5.474 | - - 1. 0.290 |
| 17 | N | 17 | H | 4.197 | 0.142 |
| 21 | N | 21 | H | −1.521 | - - 1. 0.179 |
| 29 | N | 29 | H | 1.450 | - - 1. 0.220 |
| 33 | N | 33 | H | −3.710 | - - 1. 0.209 |
| 35 | N | 35 | H | −4.014 | - - 1. 0.153 |
| 42 | N | 42 | H | 0.912 | - - 1. 0.110 |
| 44 | N | 44 | H | 2.980 | - - 1. 0.185 |
| 45 | N | 45 | H | 9.975 | - - 1. 0.077 |
| 47 | N | 47 | H | 1.885 | - - 1. 0.101 |
| 53 | N | 53 | H | 8.454 | - - 1. 0.258 |
| 54 | N | 54 | H | −0.061 | 0.368 |
| 57 | N | 57 | H | 9.975 | - - 1. 0.205 |
| 58 | N | 58 | H | 8.089 | - - 1. 0.067 |
| 59 | N | 59 | H | −10.520 | - - 1. 0.112 |
| 61 | N | 61 | H | 15.814 | - - 1. 0.378 |
| 63 | N | 63 | H | 10.218 | - - 1. 0.080 |
| 72 | N | 72 | H | 12.590 | - - 1. 0.296 |
| 73 | N | 73 | H | 2.737 | - - 1. 0.453 |
| 75 | N | 75 | H | −2.615 | - - 1. 0.272 |
| 79 | N | 79 | H | −1.825 | - - 1. 0.156 |
| 80 | N | 80 | H | −4.744 | - - 1. 0.154 |
| 81 | N | 81 | H | 3.893 | - - 1. 0.111 |
| 84 | N | 84 | H | 0.547 | - - 1. 0.423 |
| 89 | N | 89 | H | 2.980 | - - 1. 0.158 |
| 98 | N | 98 | H | 3.649 | - - 1. 0.422 |
| 100 | N | 100 | H | 13.990 | - - 1. 0.271 |
| 102 | N | 102 | H | 7.603 | - - 1. 0.203 |
| 106 | N | 106 | H | −0.791 | - - 1. 0.140 |
| 107 | N | 107 | H | −0.547 | - - 1. 0.490 |
| 108 | N | 108 | H | −1.460 | - - 1. 0.320 |
| 109 | N | 109 | H | −6.386 | - - 1. 0.119 |
| 114 | N | 114 | H | 2.798 | - - 1. 0.124 |
| 115 | N | 115 | H | −7.724 | - - 1. 0.335 |
| 121 | N | 121 | H | 0.426 | - - 1. 0.637 |
| 122 | N | 122 | H | −2.822 | - - 1. 0.243 |
| 123 | N | 123 | H | −3.528 | - - 1. 0.245 |
| 126 | N | 126 | H | 5.474 | - - 1. 0.129 |
| 130 | N | 130 | H | 4.622 | - - 1. 0.178 |
| 131 | N | 131 | H | −2.250 | - - 1. 0.327 |
| 134 | N | 134 | H | −1.885 | - - 1. 0.240 |
| 136 | N | 136 | H | −14.290 | - - 1. 0.492 |
| 143 | N | 143 | H | −11.403 | - - 1. 0.144 |
| 147 | N | 147 | H | −6.259 | - - 1. 0.251 |
| 148 | N | 148 | H | −6.386 | - - 1. 0.405 |
| 150 | N | 150 | H | −3.102 | - - 1. 0.168 |
| 152 | N | 152 | H | −1.642 | - - 1. 0.200 |
| 153 | N | 153 | H | −8.637 | - - 1. 0.500 |
| 158 | N | 158 | H | 9.427 | - - 1. 0.146 |
| 159 | N | 159 | H | −2.615 | - - 1. 0.298 |
| 160 | N | 160 | H | −10.460 | - - 1. 0.243 |
| 161 | N | 161 | H | −13.080 | - - 1. 0.452 |
| 163 | N | 163 | H | −7.481 | - - 1. 0.563 |
| 164 | N | 164 | H | −12.710 | - - 1. 0.323 |
| 166 | N | 166 | H | −4.744 | - - 1. 0.227 |
| 173 | N | 173 | H | −5.839 | - - 1. 0.243 |
| 176 | N | 176 | H | −1.703 | - - 1. 0.292 |
| 182 | N | 182 | H | −4.440 | - - 1. 0.384 |
| 186 | N | 186 | H | −2.068 | - - 1. 0.187 |
| 188 | N | 188 | H | −0.791 | - - 1. 0.112 |
| 193 | N | 193 | H | 0.791 | - - 1. 0.225 |
| 194 | N | 194 | H | −2.129 | - - 1. 0.156 |
| 195 | N | 195 | H | 0.730 | - - 1. 0.146 |
| 196 | N | 196 | H | −2.190 | - - 1. 0.207 |
| 197 | N | 197 | H | 3.345 | - - 1. 0.328 |
| 198 | N | 198 | H | 8.028 | - - 1. 0.241 |
| 199 | N | 199 | H | 1.277 | - - 1. 0.245 |
| 200 | N | 200 | H | −14.110 | - - 1. 0.694 |
| 202 | N | 202 | H | −8.150 | - - 1. 0.230 |
| 204 | N | 204 | H | −3.345 | - - 1. 0.341 |
| 205 | N | 205 | H | −7.785 | - - 1. 0.094 |
| 207 | N | 207 | H | −13.440 | - - 1. 0.258 |
| 212 | N | 212 | H | −8.211 | - - 1. 0.243 |
| 214 | N | 214 | H | −7.846 | - - 1. 0.085 |
