# Supplementary material for: Correlated Inter-Domain Motions in Adenylate Kinase
Source: PLoS Comput Biol. 2014 Jul 31;10(7):e1003721. doi: 10.1371/journal.pcbi.1003721 (PMC4117416; doi:10.1371/journal.pcbi.1003721)
Supplement: Text S1 — Supporting methods. (DOCX) [file pcbi.1003721.s020.docx]

PELE: Protein Energy Landscape Exploration.

The Protein Energy Landscape Exploration (PELE)^1^ method uses a Monte Carlo (MC) scheme where new trial configurations are produced with protein structure prediction methods. The program, originally designed to explore ligand migration in proteins, has recently been expanded to map protein conformational dynamics. PELE's heuristic algorithm is based on three main steps: *i*) ligand (if present) and protein perturbation, including the protein α-carbon displacement following an anisotropic network model approach; *ii*) side-chain sampling, by placing all side-chains local to the ligand; and *iii*) energy minimization, involving a region including, at least, all residues local to the atoms involved in *i* and *ii*. These three steps compose a movement, which is accepted (defining a new minima) or rejected based on a Metropolis criterion for a given temperature. Typically, a simulation involves several processors running multiple steps and sharing information towards addressing a common task. In this study, the task was defined as moving away and back to the initial crystal structure. The master processor keeps track of the best set of system coordinates in one (multiple) task, for example in increasing the rmsd distance from the initial structure. At the end of each cycle, each task value for the different trajectories are compared with the best stored one. If one processor lags behind, within a given threshold from the best value, it will reset its position by receiving the best stored coordinates before starting a new cycle. The simulations were based on a 12 processor PELE run. All processors shared information and drove the system in the RMSD cycle from the initial closed and open structures (1ake and 4ake), with a spawning window of 3 Å. The simulation included the 8 first anisotropic network model (ANM) modes with a maximum Calpha displacement of 1.5 Å in each backbone perturbation. The same direction, made of a 75% component of a randomly chosen mode plus 25% mixing of the remainder modes was kept for 6 iterations. Modes were recalculated every 24 iterations. Following the backbone perturbation step, the top 30 excited side chains were re-optimized (after a randomization of the initial rotamers).

*Example input file to run PELE:*

The script given below performs 7 task, combining spawning away and back to the initial structure with free exploration (for 200 steps) after the spawning tasks. The backbone perturbation uses the lowest 8 eigenmodes. Then it follows each mode (with maximum displacements of 1.5 A) for six consecutive steps. Every six steps a new random mode is chosen. Every 24 steps the modes are recomputed. Thirty side chains, the ones with a larger increase in energy along the backbone perturbation, are explicitly sampled.

To use in the server ([pele.bsc.es](http://pele.bsc.es)), choose the “Protein Local Motion” script. Select 12 processors and 48 hours. Upload the pdb file. Select Edit&Submit. Modify everything below the load pdb line in the scrip (thus, remove everything below the load line and add everything starting at the pka in the present script)

Then submit the Edited script!

--------------------------------------------------------------------------------------------------

Script:

file datadir /home/bsc72/bsc72328/plop/data

file logfile dyn.log

energy params solvent vdgbnp

energy params ionic 0.15

load pdb prep_min.pdb

pka change B:126 HIE

pka change B:134 HIE

pele &

  top_side 30 &

  init_min no &

  task &

    spawn task_num gt 0.5 &

    show rmsd_sys calpha &

    spawn ini_rmsd_sys calpha gt 3.0 &

    exit ini_rmsd_sys calpha gt 7.75 &

  task &

    exit steps gt 200 &

  task &

    spawn task_num gt 0.5 &

    spawn ini_rmsd_sys calpha lt 1.0 &

    exit ini_rmsd_sys calpha lt 1.5 &

  task &

    exit steps gt 200 &

  task &

    spawn task_num gt 0.5 &

    spawn ini_rmsd_sys calpha gt 3.0 &

    exit ini_rmsd_sys calpha gt 7.75 &

  task &

    exit steps gt 200 &

  task &

    spawn task_num gt 0.5 &

    spawn ini_rmsd_sys calpha lt 1.0 &

    exit ini_rmsd_sys calpha lt 1.5 &

  end_task &

   temp 1000 &

   wrfre 1 &

   spfre 1 &

   mifre 1 &

   anmfreq 1 &

  side &

   failsafe no &

   quiet yes &

  sideend &

   minadd beg end &

  min &

   mxitn 100 &

   rmsg 0.01 &

   nbup yes &

   alphaup yes &

  minimend &

   caconst 1.0 &

   anm_eig_freq 24 &

   anm_altm_freq 6 &

   anm_altm_type 3 &

   lanmanm neig 8 &

   lanmanm move_ca 1.5 &

   lanmanm mix_modes 0.75 &

  lanmmin &

   mxitn 100 &

   iter 1 &

   nbup yes &

   alphaup no &

   rmsg 0.01 &

  minimend

Protein samples and NMR spectroscopy for AK_e_.

^15^N enriched AK_e_ was expressed and purified following an established protocol.^2^ Backbone resonance assignments of apo AK_e_ has been accomplished previously.^2^ Steric alignment of AK_e_ was introduced with the method of stretched polyacrylamide gels. A detailed description of the experimental procedure and parameters used for measurement of NH RDCs has been reported.^3^ Errors in NH RDCs (0.3 Hz) were estimated based on the standard deviation of RDCs obtained from triplicate measurements of NH couplings in both isotropic and anisotropic phases. In Supporting Information Tab. S3 the experimentally measured RDCs are provided.

**References**

[1] K. W. Borrelli, A. Vitalis, R. Alcantara, V. Guallar, *J. Chem. Theory Comput.* **2005**, *1*, 1304

[2] J. Adén, M. Wolf-Watz, *J. Am. Chem. Soc.* **2007**, *129*, 14003.

[3] Wolf-Watz, M. et al. *Nat. Struct. Mol. Biol.* **2004**, *11*, 945-949.

Determination of distributions with two independent methods to calculate the alignment tensor.

We determined the inter-domain structural heterogeneity of AK_e_ using two independent approaches to calculate the alignment tensor: PALES and the method developed by Almond and Axelsen, herein referred as ALMOND. The two methods rendered consistent distributions (**Supporting Figs. S3** to **S5**), although some differences were observed. The two methods mostly differed in the presence/absence of a low-populated state at ca. 50º, 138º. Because ALMOND method was able to fit best the RDCs for AK_e_ the distributions obtained with this methods are presented in the main text.

Attempts at determining distributions from randomly scrambled RDCs.

In order to illustrate that the distributions obtained are driven by the experimental data we attempted to determine distributions after randomly scrambling the experimental RDCs. The results, presented in **Supporting Fig. S6**, show that it is in fact not possible to fit the scrambled RDCs to any distribution of conformations from the pool. This result shows that the distributions obtained are encoded in the experimental data and therefore are not driven by the computational approach used.

Impact of error in measured RDCs on the experimental distributions determined.

In the **Supporting Fig. S7** we present an evaluation of the impact of experimental error on the distributions determined for AK_e_ in this work. To this end, we added several levels of random Gaussian error to the experimentally measured RDCs prior to ensemble calculations, ranging from 0 to 32% of error relative to the maximum coupling observed. The distributions were found robust up to errors of *ca* 4% (0.5 Hz) and moderately robust up to 8% of error in the RDCs (1.0 Hz), a threshold that exceeds the experimental error of RDCs for this protein (~3%; < 0.5 Hz).

Impact of error in the alignment magnitude on the experimental distributions determined.

The prediction of the Saupe tensor in equation 1 (main text) requires knowledge of the absolute concentration (percentage) of the alignment medium. In the following this is addressed. First, note that because a regression method is used to scale the calculated ensemble-averaged RDCs to the experimental values, introducing error in the absolute magnitude of alignment on the ensemble averaged RDCs would be automatically taken into account by the regression method used, leaving the computational experiments unaffected. Under such circumstances, it is the error in the relative alignment between conformers that has most impact on the results. Therefore, in order to examine the effect of error in the prediction of the magnitude of alignment we devised the following test: the RDCs predicted for each conformation in the pool were scaled by a number which depended on the position along the reaction coordinate (RC) used in this work. The error was increased exponentially along the RCs. Taking the closed state as reference, scaled by an error factor of 1.0, the error factors at the end points of the RCs studied reached values of 2, 3, 10, 20 and 50-fold. The results are shown in **Supporting Fig. S8**. The distributions were found robust against errors in the prediction of the alignment magnitude within a range of 1 to 10 for both systems e.g. the alignment of the most closed and most open structures are scaled by a factor of 1 and 10, respectively. As the results show, the population most affected corresponds to that of the open state, which decreases with increasing overestimation of its alignment magnitude.

Assessing over-fitting.

In ensemble calculations over-fitting is a potential risk. Although impossible to disregard, in the following we present evidence that suggests that over-fitting is not playing a major role in the two cases studied. First, we selected an ensemble of minimum size based on both Q_work_ and Q_free_ descriptors (**Supporting Figs. S3** to **S5**). Second, we were able to qualitatively validate the ensembles against independent data. For the AK_e_ case, the ensemble determined qualitatively agrees with two independent smFRET experiments (see the main text). Moreover, the distributions converged into well defined ensembles once a minimum ensemble size, relatively small *i.e.* 4-8 members, is reached (**Supporting Figs. S3** to **S5**)

Here, we investigate this last observation in more detail. To this end, a computational experiment was performed in which we designed a unimodal and a four state distribution for AK_e_ and calculated their corresponding RDCs. These synthetic RDCs were used to rebuild the underlying distributions using the approach described in the main manuscript. Conformations used to build the target ensembles were removed from the pool of structures used to recover the distributions. We also note that while tens of structures were used for each of the basins in the designed distributions, only few conformers are required to rebuild each of the states (the ensembles calculated only contained between 1 and 64 members), clearly suggesting a predominant structural role on RDCs of different states over local details within a basin.

The results are presented in **Supporting Figs. S9** and **S10**. Converged distributions were obtained once a minimum ensemble size was reached and no deterioration of the distributions was observed for larger ensembles. Based on the agreement with free RDCs, ensembles larger than the minimum number would be selected, probably reflecting the need to account for the width of the distributions.

Reconstructing computer designed distributions in the presence of experimental error in the RDCs.

In **Supporting Fig. S11** to **S13** we present a thorough evaluation on the ability of ensemble averaged RDCs to differentiate between distributions for AK_e_ in the presence of experimental error in the RDCs. To this end, we (1) designed unimodal, bimodal, trimodal and four state distributions for AK_e_, (2) calculated their corresponding ensemble averaged synthetic RDCs, (3) added random Gaussian error to RDCs (1 to 6% of the maximum coupling value), (4) used the noise corrupted RDCs to reconstruct the underlying distributions.

Comparison of the impact of error in the alignment tensor prediction on the populations determined for AK_e_ multi-domain protein, which undergoes large inter-domain conformational changes.

To illustrate the impact of error in the alignment tensor prediction for AK_e_ protein undergoing inter-domain conformational changes of different amplitude, we have performed the following computational experiment: distributions with open and closed states populated at ratios from 0 to 100% for AK_e_ were designed. Their weighted ensemble averaged RDCs were then used to recover their underlying distributions with a pool of open and closed structures for which their alignment tensor was corrupted with increasing amounts of random error (this affected both the direction of the main alignment vectors as well as the magnitude of alignment along each of the vectors). In the **Supporting Fig. S14**, the maximum deviation observed from the correct population as a function of error in the alignment tensor is presented.
